# Use of References in Responses from Scandinavian Drug Information Centres

**DOI:** 10.3390/medicines5030066

**Published:** 2018-07-01

**Authors:** Jan Schjøtt, Ylva Böttiger, Per Damkier, Linda Amundstuen Reppe, Jens Peter Kampmann, Hanne Rolighed Christensen, Olav Spigset

**Affiliations:** 1 Section of Clinical Pharmacology, Laboratory of Clinical Biochemistry, Haukeland University Hospital, 5021 Bergen, Norway; 2 Institute of Clinical Science, Faculty of Medicine and Dentistry, University of Bergen, 5021 Bergen, Norway; 3 Clinical Pharmacology, Department of Drug Research, Linköping University, 58183 Linköping, Sweden; Ylva.Bottiger@regionostergotland.se; 4 Department of Clinical Chemistry & Pharmacology, Odense University Hospital, 5000 Odense, Denmark; pdamkier@health.sdu.dk; 5 Department of Clinical Research, University of Southern Denmark, 5230 Odense, Denmark; 6 Pharmacy Division, Faculty of Nursing and Health Sciences, Nord University, 7800 Namsos, Norway; linda.a.reppe@nord.no; 7 Department of Clinical Pharmacology, Bispebjerg University Hospital, 2400 Copenhagen, Denmark; jens.peter.konnerup.kampmann@regionh.dk (J.P.K.); Hanne.Rolighed.Christensen@regionh.dk (H.R.C.); 8 Department of Clinical and Molecular Medicine, Norwegian University of Science and Technology, 7491 Trondheim, Norway; olav.spigset@legemidler.no; 9 Regional Medicines Information and Pharmacovigilance Centre (RELIS Midt-Norge), Department of Clinical Pharmacology, St. Olav University Hospital, 7006 Trondheim, Norway

**Keywords:** adverse effects, breast feeding, complementary medicine, drug information centres, drug information sources, pharmacokinetics, pregnancy, references

## Abstract

**Background:** The aim of this study was to compare use of references in responses from Scandinavian drug information centres (DICs). **Methods:** Six different fictitious drug-related queries were sent to each of seven Scandinavian DICs. The six queries concerned adverse effects, pharmacokinetics, pregnancy, complementary medicine, polypharmacy, and breast feeding. References in the responses were categorised into five types of drug information sources: primary (original studies), secondary (reviews), tertiary (drug monographs, handbooks, etc.), DIC database, or personal communication. **Results:** Two hundred and forty-four references were used in the 42 responses. The mean number of references varied from 3.0 to 10.6 for the six queries. The largest difference between centres with regard to number of references used (range 1–17) was found for the query on complementary medicine. In total, 124 references (50.8%) were tertiary, and only 10 of the 42 responses (23.8%) did not have any tertiary references included. Complementary medicine, breast feeding, and pregnancy were query types associated with relatively frequent use of primary references. Use of DIC database was not uncommon, but personal communications were seldom used. **Conclusions:** Scandinavian DICs differ substantially in number and type of references to identical drug-related queries. Tertiary sources are mainly preferred irrespective of type of query.

## 1. Introduction

To promote rational use of medicines, drug information centres (DICs) provide evidence-based advice on therapeutic drugs in response to queries from health care professionals. The Scandinavian DICs are regional centres affiliated with units of clinical pharmacology at university hospitals. Pharmacists and physicians (clinical pharmacologists) with expertise in searching and critical evaluation of literature constitute the staff. The centres produce written responses with references to drug-related queries from health care professionals [1,2,3,4]. The queries to Scandinavian DICs are similar to other European and U.S. drug information services with frequent questions concerning psychotropic drugs, cardiovascular drugs, and anti-infectives, among others [1,2,3,4,5,6]. The queries are increasingly complex, and they frequently involve patient-related problems where decision support is needed [1,2,3,4,7,8].

The Scandinavian DICs first developed in the 1970s, even before evidence-based medicine and health technology assessment were established concepts in health care. The first DIC started in Stockholm, Sweden, in 1974, partly as a consequence of the first Medline database centre outside the USA being set up at Karolinska Institutet. The president of Karolinska Institutet at the time, Nobel laureate professor Sune Bergström, realized that the Medline system of scientific information was of major importance for both scientists and health care providers [3]. Since the 1970s, the landscape of scientific publication has changed dramatically with global, free access to PubMed since 1997, an ever-growing body of scientific publications and new methods for meta-analyses of data. Moreover, many institutions, such as the Cochrane collaboration, aimed primarily at providing evidence-based guidance to health care, have been founded [4,9,10]. There is also a growing number of predatory journals with poor quality publications [11,12].

Important for the work in the DICs is access to all types of drug information sources [13]. Sources of information in health care sciences, including drug information sources, are often categorised as primary, secondary, and tertiary [14]. Original research data presented in peer-reviewed medical journals are typical primary sources. Primary sources contain data that have not been filtered through interpretation or evaluation, other than through the peer review process. Secondary sources, such as review articles, represent interpretation of several primary sources. Thus, a secondary source evaluates, summarizes, or reorganizes information reported by researchers in the primary literature. Tertiary sources consist of information representing a further distillation and collection of primary and secondary sources. In the case of drug monographs, like the Summaries of Product Characteristics (SmPCs), information is often structured in sections or chapters addressing particular qualities (e.g., drug interactions, adverse effects, pregnancy) of a pharmaceutical product. Additional sources of information for DICs are their own databases of previous questions and answers, like Drugline in Sweden and the RELIS database in Norway [2,3].

The International Pharmaceutical Federation (FIP) has recommended that DICs should perform quality assurance activities, such as regular review of responses and periodic review of resources and procedures [15]. There is no formal international consensus concerning the use of references in responses from DICs today [16]. However, the Scandinavian DICs still work according to the same protocol as was set up in the 1970s, with structured literature searches and evaluation of published data in relation to the clinical query at hand [4]. Thus, it is of interest to compare the use of references in responses to drug-related queries from Scandinavian DICs as a basis for discussions on quality assurance of the work performed.

## 2. Materials and Methods

### 2.1. Study setting

Eleven Scandinavian DICs were presented with the protocol for the study, and seven chose to participate; four Norwegian, two Danish, and one Swedish centre. The main reason for not participating was a reported lack of time and resources. Six fictitious queries typical of those usually received by DICs in Scandinavia, and similar to queries most often registered by DICs in other countries, were used [1,2,3,4,5,6,7,8]. The queries were based on the experience that physicians often ask extremely focused questions with clinical and laboratory diagnostic information, but where important information could be withheld [8]. The queries had no clear-cut answers and comprised the most common query categories that DICs respond to [1,2,3,4,5,6,7,8]; namely, adverse effects, pharmacokinetics, drug use in pregnancy, polypharmacy, complementary medicine, and drug use during breast feeding. Five queries were patient-specific and one had a more general perspective (Table 1).

We recruited general practitioners (GPs) familiar with each of the DICs to pose the six queries by e-mail to each centre. Some queries had a requested response time. Queries were originally asked in Norwegian, Danish, or Swedish according to the location of the centre. The GPs asked each query to “their” centre on the same day during a total period of eight weeks during January–March 2013.

Staff members at the participating centres were informed about the study, but were blinded in terms of which queries were the study queries. Details of the project design have been presented previously [17,18].

### 2.2. Analysis of references

References in the responses were categorised into five types of drug information sources; primary (original studies), secondary (reviews), tertiary (drug monographs, handbooks, etc.), DIC databases (use of previous responses), or personal communication. Only references in the written responses that addressed statements in the text, and were organised in a reference list, were included. Other sources mentioned in the responses, like enclosed articles or tips for supplementary readings on a subject, were not included. Furthermore, statements concerning the search strategy employed (e.g., “we consulted the following sources”) with negative results (not further mentioned in the text) were not defined as references in the study. Use of previous response from own DIC database and personal communications were included to assess the use of these options, which is of relevance to Scandinavian DICs.

### 2.3. Statistics

Descriptive statistical analyses were performed using Statistical Package for the Social Sciences (SPSS) version 24.0 (IBM Corp., Armonk, NY, USA, 2016)

## 3. Results

Two hundred and forty-four references were used in the 42 responses, with substantial differences in the mean number of references (3.0–10.6) used to answer the six queries (Table 1). The overall mean number of references was 5.8. The highest number of references was used in the polypharmacy query, where four of the seven DICs used ≥10 references, whereas the lowest number was used in the pregnancy query, where five of the seven DICs used ≤2 references. The query with a requested time frame for the response of only one day had fewer references included (mean 3.0) than the other questions, for which there were less time constraints (means 4.7 and higher) (Table 1).

In total, 124 references (50.8%) were tertiary (Table 2), and only 10 of the 42 responses (23.8%) did not have any tertiary references included. For the polypharmacy query, 61/74 references (82.4%) were tertiary. Complementary medicine and breast feeding were queries associated with relatively frequent use of primary references (Table 2).

**Figure 1:**
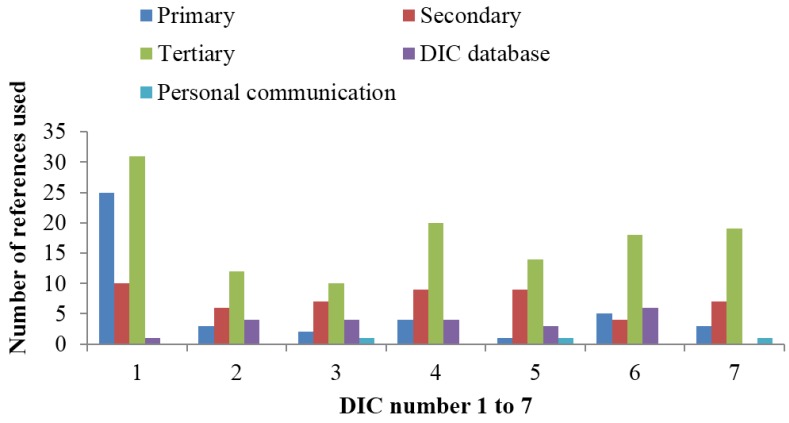
Number and type of references used by the seven Scandinavian drug information centres (DICs) participating in the study.

**Figure 2:**
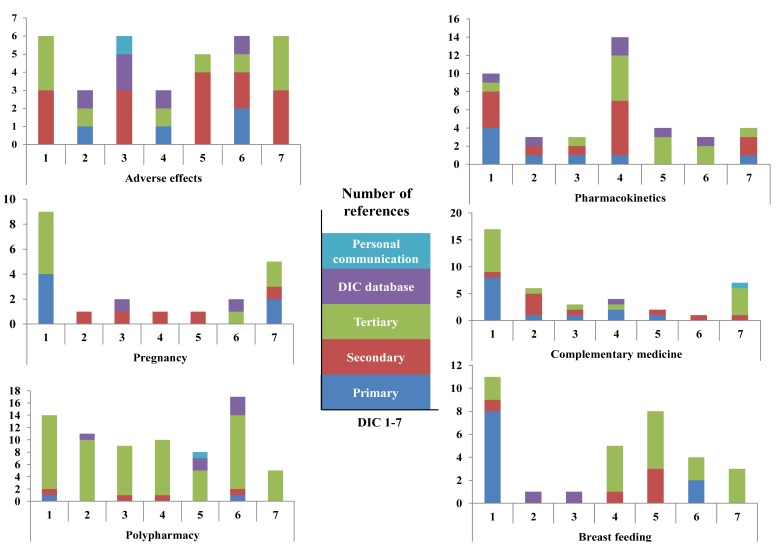
Type of references related to type of query for the seven Scandinavian drug information centres (DICs) participating in the study. Note that the ordinates are displayed with different scales.

Overall use of references in the individual DICs is presented in Figure 1. For all DICs, tertiary references were the most commonly used type. One DIC used substantially more references than the other six, and this centre had a proportion of primary references that was more than twice that of the other centres. Use of own DIC database as a source was not uncommon, while reference to personal communications occurred only three times in the study. Figure 2 shows the use of references for each of the seven DICs with regard to the type of query. The largest difference between the centres with regard to the number of references (range 1–17) was for the query concerning complementary medicine. For the polypharmacy query, all of the DICs used more references than for the other queries.

It was difficult to find any consistent pattern between or within DICs with regard to use of references to different types of queries. Great diversity in use of particularly primary and secondary references was generally observed. However, in response to the query concerning adverse effects, a position paper concerning the rare, but serious adverse drug reaction of bisphosphonate-related osteonecrosis of the jaws, either as an article [19] or available as a pdf from the American Association of Oral and Maxillofacial Surgeons’ website [20], was cited by four out of seven DICs. The references used for the query concerning pregnancy showed that the two Danish DICs used different primary references, the four Norwegian DICs used the similar secondary reference (a review in Norwegian), while the Swedish centre used a previous response from their own DIC database. In general, national (and language) preferences were found in the use of secondary references (e.g., reviews), tertiary references (drug interaction databases), or use of DIC databases. Furthermore, national subscriptions (e.g., access to Natural Medicines database) not available for all seven DICs influenced the use of references. However, because of the close relationships between the Scandinavian languages, some DICs used references written in another Scandinavian language as well.

## 4. Discussion

The main finding in this study was that Scandinavian DICs differed substantially in use of number and type of references to identical drug-related queries and use of tertiary sources dominated, irrespective of type of query. For questions concerning complementary medicine, breast feeding and pregnancy primary sources were also common.

Queries to Scandinavian DICs are frequently consultative with clinical problems and less often factual (e.g., “what is the half-life of tramadol?”) [1,2,3]. The response time is agreed upon through a web-based query form, e-mail, or on the telephone. If requested, a preliminary answer is followed by a written response with references. Twenty years before the present study, drug information sources used to answer 461 consecutive queries to one Scandinavian DIC were retrospectively assessed [21]. The authors found that primary sources were most frequently used (36%), followed by the DIC database (31%). Typical tertiary sources such as textbooks (e.g., Martindale, Meyler’s Side Effect of Drugs) represented 18.9% of references. In comparison, 51% of references in the present study were tertiary, and only 9% were from own database. A single centre answered all of the queries in the 1993 study, while the present study involved seven centres. In addition, the present study concerned six different questions (a total of 42 answers), as compared with 461 responses in the 1993 study. Thus, direct comparisons are difficult. However, the two studies, although methodologically different, were performed in a similar context. The three most common categories of queries in the 1993 study were adverse effects, pregnancy, and therapeutic use of drugs, and these are still among the top categories of queries to Scandinavian DICs [1,2,3,4].

There has been a significant development in technology, workload, and organisation of DICs in the last twenty years. The most extensive change in drug information sources concerns tertiary sources with electronic accessibility and improved user-friendliness for literature search. A recent survey of pharmacies in the United States found frequent use of tertiary sources [22]. About 89% of community pharmacists and 96% of hospital pharmacists reported that available tertiary references were adequate to answer the questions they received. Use of tertiary sources is also a recommended starting point for search strategies for pharmacists [16,23]. The present results suggest that tertiary sources are the most important drug information tool in DICs as well, which at least in part could be related to improvements in their quality and accessibility in the last decades. 

Use of primary (and secondary) sources has not changed during the last twenty years, and utilization of these sources requires critical literature evaluation skills in the staff. Our group has previously shown that type of literature search is of particular importance for the time spent handling queries to DICs [24]. An extensive search for original articles can be time consuming, and will also increase the time needed for reading and analysing the studies. The increased use of tertiary sources could also be related to a higher workload in the DICs with an increasing number of queries, in addition to the steadily increasing total amount of drug information available [4]. The impression from the present results is that use of primary sources was dependent on the individual staff member handling the query, while national preferences, DIC preferences, and availability of sources (subscription), among others, influenced the use of secondary and tertiary sources.

Availability of user-friendly and quality-assured tertiary sources could make the DICs more effective. Interestingly, from 1993 to 2013, the mean number of references per answer increased from 2.8 to 5.8. Mean time consumption responding to queries posed to the Scandinavian DICs in 1990 and in 2013 was 187 and 178 min, respectively [25,26]. In a previous study, we found that time consumption increased as the number of sources searched increased, with contradicting information in various sources of information and with difficulties finding documentation on the specific question [24]. We have also previously reported that the quality of the 42 responses assessed in the present study was generally good-to-very-good, and that time consumption and quality were only weakly associated in this setting [17]. In that study, clinical pharmacologists (internal experts) and general practitioners (external experts) reviewed responses individually. The association between time consumption and total quality of the responses did not reach statistical significance when adjusted for response time [17]. In a related study from our group, medical and language experts examined the 42 responses included in the present study [18]. With a few exceptions, the experts also judged the pharmacological content in the responses to be concordant. The responses to the same query grossly differed in some instances in the number and types of references included, and some experts commented with the term “weak” and/or “thin” references. There were different opinions within the medical expert group as to whether it was sufficient to use secondary/tertiary resources and formerly answered queries to produce responses, or whether primary articles should be used. However, none of the experts explicitly stated that they preferred the use of primary references [18]. In an earlier study of the Norwegian DICs, evaluation forms accompanying 163 written answers to physicians were used to examine the quality and impact of the responses [27]. About 80% of the physicians found that the response was sufficiently fast and that the answer was relevant, adequately comprehensive, and with valuable references, while an additional 16% found that three of these four quality criteria were satisfied [27]. There was naturally a great variation in the type and number of references in these 163 written answers. Thus, it is difficult to extract “a best practice answer” from these studies with regard to use of references. The U.K. Medicines Information Centres’ “Guide to writing medicines Q & A’s” and checklists for quality assurance of queries and answers are available online [28]. Included in the checklist is that no mistakes should be made in the referencing, and that a primary literature search (including the use of Embase/Medline) should always be performed [28].

It is not possible to compare the quality of the responses from 1993 and 2013, but the results from studies involved in the present project suggest that use of appropriate and credible sources, along with critical literature evaluation skills, may be more important than exactly what type of source is used [17,18]. Critical literature evaluation is reflected in a careful use of previous answers from the DIC database. Furthermore, the experience and competence in the DICs are reflected in the minor use of reference to personal communications.

In the present study, primary sources appeared especially useful in queries concerning drug use in pregnancy and breast feeding. A reason for this could be that tertiary sources, for example, product monographs, are typically very categorical on these subjects, and drugs are often classified according to a risk evaluation predominantly based on animal studies [29]. Moreover, the pharmaceutical industry is focused on disclaimers and restrictive attitudes in their monographs because of legal considerations, and such an approach is counterproductive for clinical decision support. Use of previous responses can be of particular value for DICs, and DIC databases with previously answered queries are regarded as an important resource for Scandinavian DICs [3,4]. In several of the queries included in the present study, some DICs had recently answered similar queries (quaternary sources), and these previous responses gave quite clear recommendations in terms of, for example, drug use in pregnancy and breast feeding. In a recent study of queries concerning breast feeding to Norwegian DICs, about 40% of the queries used original articles or review articles [30].

The use of primary sources in the query concerning complementary medicine is not surprising as the amount of tertiary sources on this subject is scarce. Furthermore, the few sources that exist usually present results from in vitro or animal studies with unclear clinical relevance [31]. Thus, it is important to search primary or secondary sources for clinical documentation. A study of queries about complementary medicine to Norwegian DICs found that a lack of relevant information represented a problem to answer complex patient-specific questions [32]. In that study, 32 out of 100 responses used original articles (primary and secondary sources) from searches in databases like Medline, Embase, and Cochrane. In our experience, complementary medicine is less used in Scandinavian countries when compared with the rest of Europe and also with other parts of the world, and we do not know whether DICs elsewhere, with more frequent queries of this category, handle them more efficiently with access to other sources. In contrast, breast feeding is highly advocated in our countries, and because of frequent queries about breast feeding (and/or pregnancy), we have experience and quality sources to provide answers, as discussed above [30].

In the question concerning polypharmacy, use of tertiary sources dominated among all the DICs. Polypharmacy often concerns drug interactions, and with these queries, drug interaction databases are useful. In most of the recommended sources, it is possible to submit a large number of concurrent drugs to check for interactions in an efficient way. We have previously shown that the number of drugs in the query does not influence the time spent answering it [20,22]. However, choice of drug interaction databases is of importance as open access drug interaction databases score statistically lower with regard to ownership, classification of interactions, primary information sources, and staff qualification [33].

According to textbooks on drug information resources for pharmacists, tertiary sources represent a good starting point for drug information searches [16,23]. The use of tertiary sources, like drug monographs with structured chapters, is, in our experience, ideal in telephone services where immediate answers are required. This is also an opportunity to gain general information about the disease or drug in question, which will ultimately result in a more structured and productive search through secondary and primary sources. Tertiary sources can be used to provide a preliminary answer, before other sources are consulted. As references and not searches were compared in the present study, we cannot answer whether it is always necessary to consult primary sources before providing a response. However, the present results suggest that DICs are more eclectic in choice of searches based on type of query. We believe this is linked to the present workload and time consumption in DICs, the technical and user-friendly level of tertiary sources, the possibility of re-use of previous answers, and the communication between DICs with exchange of information [34].

A relevant question in a historical perspective is whether DICs today are less evidence-based since the use of tertiary references has increased. The present study cannot answer this question conclusively. However, tertiary references have developed substantially since the 1970s, and there is substantial criticism of primary and secondary sources. Development of misleading and conflicted systematic reviews and meta-analyses and the large amount primary (and secondary) literature of low quality today is a challenge [4,9,10,11,12]. Importantly, DICs do not develop guidelines, but relate guidelines to provide decision support for rational pharmacotherapy of individual patients. This important role of DICs is a reflection of the weakness of many current guidelines, which often concern one disease and lack recommendations for clinical management at the patient level. In this respect, DICs contribute to personalized medicine or precision medicine to tailor therapy with the best response and highest safety margin to ensure better patient care. According to our experience there is an increasing number of queries concerning genotyping, either on a more general basis or related to a specific polymorphism (e.g., in a drug-metabolising enzyme, a transporter, or a receptor) diagnosed in a patient.

### Limitations

We posed only six queries to the DICs during this study. Although these were typical for the centres included, the responses may not be representative for all the responses given. Nevertheless, the importance of this study does not necessarily lie in the quality of each response, but in the possibility to compare different responses with the same query, and therefore control the responses against each other. As mentioned above, references and not searches were used to compare the DICs. The number of references in the responses does not necessarily reflect the extent of search performed or the search strategy. However, selection and precise use of references in responses from DICs is regarded as an important quality measure by medical and language experts [18]. The Scandinavian countries have similar traditions for drug therapy and drug information, and we did not expect different DICs to give professionally different responses. We knew, however, that the centres had different writing styles, working procedures, and structure of the responses. In this small study, no specific DIC’s style stood out in terms of providing better quality responses than the others with regard to use of references.

## 5. Conclusions

Access to updated tertiary sources is of importance for Scandinavian DICs. Moreover, such sources seem to be useful for most types of queries. Use of primary and secondary sources is linked to type of query, in particular, when tertiary sources are categorical, controversial, or lacking. In a historical perspective, the importance of primary sources as references is reduced in the DIC setting, which could be related to factors such as the technological development of and accessibility to high quality tertiary sources, total workload in the centres, and skills among the staff.

## Figures and Tables

**Table 1:** Type of query with total, mean, and range of number of references among the seven Scandinavian drug information centres (DICs) participating in the study.

Category	Query	Requested Response Time	References		
			Total	Mean	Range
Adverse effects	A female patient presents with deep, infected pockets close to the jaw bone, and needs to have these rinsed every fourth to fifth week for the next six months. The patient uses alendronate 70 mg once weekly. Should alendronate be discontinued during treatment?	Within a week	35	5.0	3–6
Pharmacokinetics	A male patient with formerly performed gastric bypass needs treatment for *Helicobacter pylori* infection because of symptomatic ulcus. The GP wants to start treatment with pantoprazole 40 mg once daily, metronidazole 400 mg twice daily, and amoxicillin 750 mg twice daily. Will the absorption of the drugs be reduced, and are dosage adjustments necessary?	Within 2 days	41	5.9	3–14
Pregnancy	A pregnant women manifests with moderate depression (MADRS^1^ score 29), and there is indication for treatment with an antidepressant. What antidepressant is the first choice of drug during pregnancy?	Within the next day	21	3.0	1–9
Complementary medicine	A GP has registered an increasing use of Ginkgo biloba in nursing care homes and home nursing services. He (she) has also registered that ginkgo might increase bleeding time. What documentation exists on this topic, and what is the relevance for concomitant use of, for example, warfarin, acetylsalicylic acid, clopidogrel, and enoxaparin?	None	40	5.7	1–17
Polypharmacy	A male patient, 75 years old, has gradually developed impaired cognition for the last five to six months (MMS^2^ score 18 at examination). He has essential hypertension treated with atenolol 100 mg once daily and losartan/hydrochlorothiazide 100/12.5 mg once daily. His blood pressure was 130/90 mmHg at the latest appointment. He also uses simvastatin 40 mg and acetylsalisylic acid 160 mg once daily. He uses paroxetine 40 mg in the morning for anxiety/depression, diazepam 5 mg as needed, and promethazine/propiomazine^3^ 50 mg at night for sleep. He also uses tolterodine 2.8/4 mg^4^ once daily for overactive bladder (dosage increased from 1.4/2 mg^4^ three months ago). The patient does not smoke. Can any of these drugs, or drug interactions, increase the risk of impaired cognition?	None	74	10.6	5–17
Breast feeding	A female patient, 13 weeks post partum, presents with active ulcerative colitis. She has earlier been treated with sulfasalazine 500 mg × 3 (discontinued during pregnancy). Can she use sulfasalazine while breast feeding?	None	33	4.7	1–11
**Total**			**244**	**5.8**	**1–17**

The six drug-related queries from general practitioners (GPs) were simultaneously submitted to the seven DICs during a study period of eight weeks. All GPs asked each query on the same day. Queries were originally asked in Norwegian, Danish, and Swedish languages. Elements in this table have previously been published in another article from our group (17). ^1^MADRS, Montgomery and Asberg Depression Rating Scale. ^2^MMS, Mini Mental Status. ^3^Promethazine is marketed in Norway and Denmark, but not in Sweden. For the Swedish query, we used the drug propiomazine. Both drugs are derivates of phentiazines with antihistaminic and anticholinergic effects. ^4^Tolterodine is marketed as 2 and 4 mg depot capsules in Norway and Sweden, and as 1.4 and 2.8 mg depot capsules in Denmark.

**Table 2:** Type of reference related to type of query for the seven Scandinavian drug information centres (DICs) participating in the study.

Type of reference	Total n (%)	Type of query and number of references					
		Adverse effects	Pharmacokinetics	Pregnancy	Complementary medicine	Polypharmacy	Breast feeding
Primary (original studies)	43 (17.6)	4	8	6	13	2	10
Secondary (reviews)	52 (21.3)	15	14	5	9	4	5
Tertiary (drug monographs, handbooks, etc.)	124 (50.8)	10	13	8	16	61	16
DIC database	22 (9.0)	5	6	2	1	6	2
Personal communication	3 (1.2)	1	0	0	1	1	0
**Total**	**244 (100.0)**	**35**	**41**	**21**	**40**	**74**	**33**
